# The Causes of the Deposition of Salivary Calculus

**Published:** 1872-08

**Authors:** E. C. Chase

**Affiliations:** Iowa City, Iowa.


### ARTICLE VII.

### *The Causes of the Deposition of Salivary Calculus.*

BY E. C. CHASE, D. D. S., IOWA CITY, IOWA.

With the exception of a few French authors, all the writers of dental literature agree in the well established fact that the source or origin of salivary calculi is in the saliva. Taking this for granted then, viz: that the elements, not necessarily the proximate principles, that are found in tartar —I have used the two terms, tartar and salivary calculus, synonymously—are the components of the insoluble or inorganic portion of the saliva, we will proceed to demonstrate how and why they are precipitated from it. First let us inquire as to the chemical constitution of the inorganic principle of the saliva.

The fluid not being a fixed secretion, but depending in a great measure upon the physical condition of the system regarding the quantity and quality secreted, it is therefore not at all strange that there should be found such great variations in the analyses that have been made. By comparing several analyses made by the best authorities, and averaging them, we find that about five per cent. of the mixed saliva consists of solid matter; now deducting two per cent. for mucus and epithelium, we have three per cent., consisting principally of phosphate of lime, free soda, and the alkaline lactates of lime, soda, and potassa, but observe no *carbonates;* whereas in most cases tartar or salivary calculus is composed of from ten to twenty per cent. of carbonate of lime; now from whence is this substance derived, it not being found in the saliva? We shall try to account for its presence before we are through.

The above, then, may be considered a fair statement of the chemical constitution of the solid portion of the saliva.

Now let us look for a moment to the chemistry of tartar. We find that the most reliable analyses gives from sixty to seventy-five per cent. as strictly inorganic matter, the balance being made up of mucous, fibrin, epithelium, and water. The inorganic portion is that with which we have to deal.

Now comes the main question, and in fact, the only one that is suggested by the heading of this article, viz : how is the phosphate ot lime precipitated, and what is the origin and cause of the deposition of the carbonate of lime that is found to compose a large per cent. of tartar, but which does not exist in the mixed saliva.

As to the first part of the question, we have seen that the phosphate of lime exists as phosphate of lime in the saliva, hence it is simply precipitated from that fluid, undergoing no chemical change. The saliva from all the glands, the parotid, the sub-maxillary, and sub-lingual, while being secreted, and while passing from the mouths of their respective ducts is normally alkaline, owing to the presence of the alkaline lactates and the free soda, but after it has once become mixed, or even as soon as it becomes exposed to the air it is immediately changed from an alkaline to a neutral or slightly acid fluid, which can easily be proved by any one with litmus paper. This change may be brought about in two ways : First, the acids resulting from the putrefactive decomposition of particles of food or epithelium, the presence of which is the chief, if not the only cause of dental caries, coming in contact with the saliva would instantly unite with the free soda, and if powerful enough, decompose the lactates, setting free lactic acid and uniting with the bases, forming salts ; thus if hydrochloric acid were present there would be formed the chloride of calcium, sodium, and potassium, with the liberation of lactic acid.

Second, or the neutrality or acidity might be brought about by the carbonate acid which passes off from the system in large quantities by each exhalation of the air from the lungs. This coming in contact with every portion of the saliva as soon as it is emptied into the mouth, would tend to transform it immediately to an acid fluid, and particularly would this be the case in the mouth of one who exhaled through this cavity instead of the nostrils.

The saliva now being changed chemically, the condition favorable to the solution of phosphate of lime of course are not present, and this substance is precipitated as an insoluble powder. It now finds its way between the margin of the gums and necks of the teeth, and is slowly but constantly deposited, first in the interstices of the enamel, and thus gets a firm hold, and then layer upon layer is piled on, until in time, if nothing interferes, there is formed a huge mass, which becomes the seat of disease and corruption, and even of animal life—infusoria.

Now concerning the latter part of the question, from whence is the carbonate of lime? We have already seen that the mixed saliva is slightly impregnated with carbonic acid coming from the lungs; this then would act at once upon the lactates of lime, soda, and potassa, forming carbonates of them all, and we would have carbonate of lime, carbonate of soda, and carbonate of potassa.

Now it is well known by all who studied chemistry that the carbonates of the alkali metals, such as potassium, iodine, ammonium, are soluble, while the carbonates of the alkaline earths, such as calcium, strontium, and barium, are insoluble. The carbonate of lime belonging to this latter class, and therefore insoluble, would be precipitated as a white powder, and like the phosphate of lime, would lodge in all protected places on the teeth, and with it make up the bulk of tartar as found in the human mouth.

During sleep the conditions are most favorable for its rapid formation; it is then that the parts are in almost perfect rest, hence it remains where deposited; now it is that respiration proceeds through the mouth, and the supply of carbonic acid is abundant.

From the foregoing will be seen the importance of preserving the normal alkalinity of the saliva, for upon this property depends its power of retaining in solution the principle ingredient of tartar or salivary calculus.—*Missouri Dental Journal.*

